# Unpaired intra-operative OCT (iOCT) video super-resolution with contrastive learning

**DOI:** 10.1364/BOE.501743

**Published:** 2024-01-16

**Authors:** Charalampos Komninos, Theodoros Pissas, Blanca Flores, Edward Bloch, Tom Vercauteren, Sébastien Ourselin, Lyndon Da Cruz, Christos Bergeles

**Affiliations:** 1 School of Biomedical Engineering & Imaging Sciences, King’s College London, SE1 7EU, London, UK; 2 Moorfields Eye Hospital, EC1V 2PD, London, UK; 3 Institute of Ophthalmology, University College London, EC1V 9EL, London, UK

## Abstract

Regenerative therapies show promise in reversing sight loss caused by degenerative eye diseases. Their precise subretinal delivery can be facilitated by robotic systems alongside with Intra-operative Optical Coherence Tomography (iOCT). However, iOCT’s real-time retinal layer information is compromised by inferior image quality. To address this limitation, we introduce an unpaired video super-resolution methodology for iOCT quality enhancement. A recurrent network is proposed to leverage temporal information from iOCT sequences, and spatial information from pre-operatively acquired OCT images. Additionally, a patchwise contrastive loss enables unpaired super-resolution. Extensive quantitative analysis demonstrates that our approach outperforms existing state-of-the-art iOCT super-resolution models. Furthermore, ablation studies showcase the importance of temporal aggregation and contrastive loss in elevating iOCT quality. A qualitative study involving expert clinicians also confirms this improvement. The comprehensive evaluation demonstrates our method’s potential to enhance the iOCT image quality, thereby facilitating successful guidance for regenerative therapies.

## 1. Introduction

Regenerative therapies [1,2] are considered promising treatments for degenerative eye diseases such as Age-Related Macular Degeneration (AMD) [3]. AMD is a leading cause of visual impairment for elderly individuals in developed countries [4]. The effectiveness of these therapies heavily relies on the precise delivery of novel therapeutic agents, either sub-retinally or intra-retinally. Consequently, robotic systems accompanied by exceptional visualization capabilities may provide the level of precision required during the implantation [5]. Intra-operative Optical Coherence Tomography (iOCT) can significantly assist such vitreoretinal surgeries by capturing cross-sectional images of the retina to guide therapy delivery.

In the pre-operative setting, Optical Coherence Tomography (OCT) is widely used to non-invasively visualize distinctive retina layers offering valuable diagnostic capabilities. OCT systems use light in the near-infrared spectral range to capture cross sectional images and subsequently apply spatiotemporal signal averaging to provide OCT images of high quality. This enables clinicians to effectively differentiate between various retinal layers and pathologies. However, the long acquisition time of pre-operative OCT scans renders them unsuitable for visualization of real-time interventions. On the other hand, iOCT provides the advantage of real-time acquisition of cross sectional retinal images in 2D and 3D with micrometer resolution. Studies indicate that iOCT can assist the precise delivery of the therapeutics in the subretinal space [6]. Nonetheless, this real-time acquisition is provided at the expense of compromised image quality as evidenced by high levels of speckle noise [7] and low signal strength [8], thereby limiting its interventional utility. The purpose of our work is to augment the capabilities of the current iOCT systems by computationally enhancing the iOCT image quality without requiring costly hardware upgrade.

Several approaches have been proposed for OCT image quality enhancement including diffusion-based [9], registration-based [10] and segmentation-based [11] methods along with Wiener filters [8], non-local filters [12] and sparse coding [13]. These methods can successfully enhance the OCT image quality by augmenting retinal layer spatial details and mitigating speckle noise, while preserving the image content. However, they rely solely on information from a single iOCT image without employing a learning-based mapping to a high-quality domain. This strategy might not be sufficient for simultaneous noise reduction and generation of high-frequency information and finer details which are vital for enhancing the overall quality of the denoised images. Furthermore, the computational cost and the long scan acquisition time prohibits the use of similar approaches for real-time iOCT quality enhancement. "Super-resolution" and "quality enhancement" are used interchangeably as usual in the literature.

Generative models such as Generative Adversarial Networks (GANs) [14] have been successfully used in image quality enhancement or image domain translation tasks using natural image datasets [15–17]. Moreover, these models have been applied to various medical image modalities such as PET [18], CT [19], OCTA [20,21], OCT [22–24] and iOCT [25–27]. However, many of these methods [20,21,24] artificially generate paired LR and HR datasets by simulating the degradation process with conventional filters. It is important to acknowledge that the super-resolution challenge we address in our problem involves a more substantial gap between LR and HR domains. This is because iOCT images are acquired in real-time during surgical procedures, which can lead to dynamic changes in tissue conditions, lighting variations, motion artifacts and high noise levels. These factors can introduce substantial variations in image appearance and quality creating a domain that can not be easily simulated by conventional filters. Also, iOCT images exhibit surgical instruments interactions, a characteristic absent in pre-operative OCT diagnostic images. Furthermore, the existing methods focus on single-image quality enhancement disregarding temporal consistency and thereby leading to inhomogeneous enhancement when applied in videos. While numerous works have been proposed for video quality enhancement [28], they necessitate well-aligned input and target videos, a condition which often remains unmet in medical imaging domain, particularly in iOCT.

This research explores unpaired iOCT video super-resolution with the objective of improving image quality of low-resolution (LR) iOCT video frames by leveraging information from high-resolution (HR) pre-operatively acquired OCT images. We propose an adversarial framework which contains a bidirectional recurrent neural network (VSR model) as generator to ensure temporal consistency, trained with a multilayer, patchwise contrastive loss to enable super-resolution between unpaired LR and HR datasets. Our VSR model adopts critical components from state-of-the-art VSR models such as BasicVSR [29] for efficient alignment and fusion of temporal information. The vast majority of the proposed VSR models (including BasicVSR) are trained on artificially paired video datasets using pixel-level supervision. As simultaneous acquisition of real LH and HR iOCT videos is not possible, we ease the requirement of pixel-level supervision by using contrastive loss. Patchwise Contrastive Learning [30] enables unpaired super-resolution by preserving the content using maximization of the mutual information between corresponding LR and generated HR patches. To establish the effectiveness of the proposed framework we provide extensive quantitative analysis showcasing our model’s superior performance against current state-of-the art iOCT super-resolution techniques. Additionally, to further support our design choices, we conducted ablation studies, revealing that the aggregation of temporal information and the use of multilayer patchwise contrastive loss play crucial roles in image quality enhancement and structure preservation, respectively.

Therefore, our contributions can be summarized as: First, we have successfully achieved a new state-of-the-art performance in iOCT super-resolution, thereby augmenting the capabilities of this imaging modality. Second, we propose a novel methodology for training VSR models in unpaired setting enabling the application of these models in domains where paired datasets are limited, such as the medical imaging domain. Also, to facilitate the super-resolution research in Optical Coherence Tomography and to provide the broader research community with a valuable resource for experimentation and further advancements, our source code will be available online at: https://github.com/RViMLab/BOE2023-iOCT-video-super-resolution.

## 2. Methods

In this section, we present the clinical data utilized in our study and we introduce our proposed unpaired video super-resolution framework. We outline its design and highlight its key features, providing implementation details for a comprehensive understanding of the methodology.

### 2.1 Datasets

**Fig. 1.**
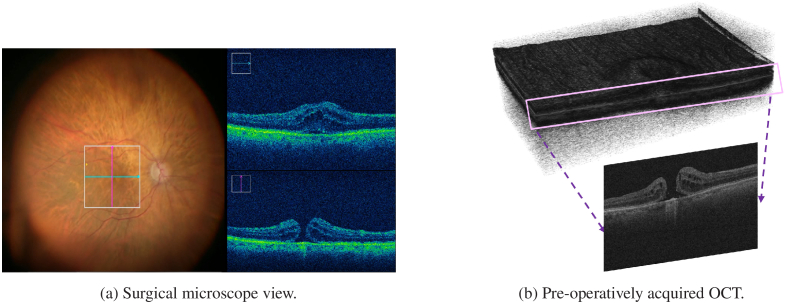
Dataset overview: (a) Microscope view during vitreoretinal surgery accompanied with low-resolution (LR) intra-operative OCT video frames. (b) Pre-operatively acquired OCT 3D volume and 2D high-resolution (HR) preOCT image. Unpaired video super-resolution is performed between iOCT videos and preOCT images.

We used an internal database which contains intra-operative videos and pre-operative OCT data accompanied with vitreoretinal surgery videos (see Fig. 1). The dataset encompasses a diverse cohort of 61 patients presenting various pathologies including: Macular hole, Epiretinal membrane, Optic disc pit maculopathy, Floaters, Choroideremia, Myopic foveoschisis, Vitreomacular traction and Synchisis Scintillans. The data acquisition process adhered to the principles outlined in the Declaration of Helsinki (1983 Revision). The intra-operative OCT (iOCT) video sequences used in our research consist of 5 frames per sequence, with a resolution of 304 × 448 pixels. Each iOCT frame has a variable width of 3–16 mm and height of 2mm. These sequences were acquired during the vitreoretinal surgeries using RESCAN 700 mounted on the Zeiss OPMI LUMERA 700. The patients’ pre-operative OCT (preOCT) scans (resolution of 512 × 1024 × 128 voxels), acquired using a Cirrus 5000, undergo a process of extraction, leading to the creation of 128 two-dimensional (2D) images. Each 2D image has width of 6mm and height of 2mm. The extracted 2D images subsequently serve as constituents of the high-resolution (HR) domain.

The dataset, 9676 iOCT video sequences and 7808 preOCT images, was split randomly into: training set (43 patients, 70%), test set (9 patients, 15%) and validation set (9 patients, 15%). The data of each patient was used only in one set. The various pathologies present in the dataset are representatively distributed between both the training and test sets, thus maintaining a satisfactory level of pathological diversity across the partitions. Please note that the data can be provided to interested researchers via a data-sharing agreement, while there are parallel endeavours to make it public.

### 2.2 Unpaired video super-resolution

The task addressed in this work is unpaired video super-resolution of iOCT. Given a LR iOCT video sequence $x\in X\subset R^{T\times H\times W\times C}$ , with T being its temporal extent, H and W being height and width respectively, C denoting its channels ( C=1 ) and each frame denoted by $x_{t}$ (of shape H×W×1 ), the goals of our video super-resolution approach are three-fold:

First, $\forall x_{t}$ we aim to generate a high resolution image ${\hat{y}}_{t}$ with the appearance and image quality of the target domain $Y\subset R^{H\times W\times 1}$ . Second, temporal information from previous input frames $\left\{{\hat{x}}_{t-N},\ldots ,{\hat{x}}_{t-1}\right\}$ , within a certain time window of size N<T , must be efficiently incorporated to generate image ${\hat{y}}_{t}$ of enhanced quality. Third, ${\hat{y}}_{t}$ must retain the structural information contained in the original LR iOCT input $x_{t}$ . We now discuss how each of those objectives is addressed by the proposed approach.

### 2.3 Appearance mapping using adversarial training

**Fig. 2.**
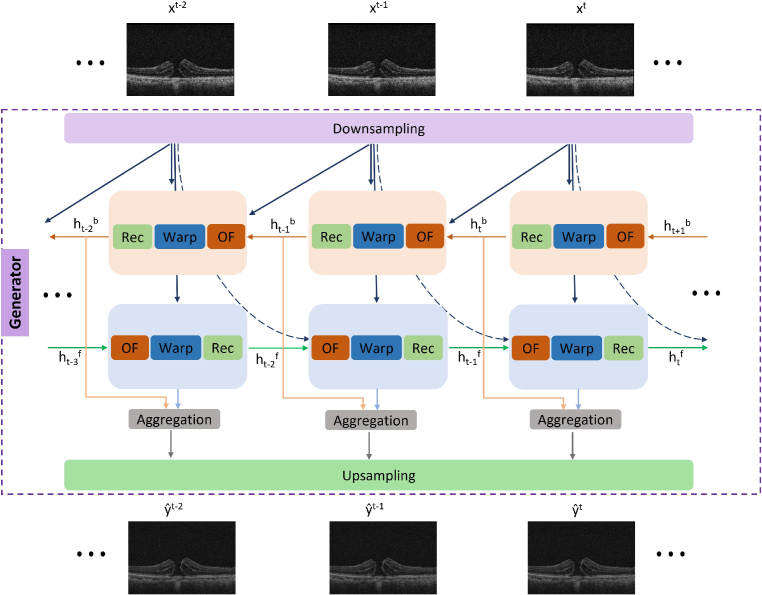
The architecture of the Bidirectional recurrent VSR network used as generator in adversarial training. The input LR iOCT sequence $x_{\left[t-N:t\right]}$ passes through: downsampling, optical flow, warping, reconstruction, aggregation and upsampling modules both in backward and forward direction generating HR ${\hat{y}}_{\left[t-N,t\right]}$ output.

To enforce that the appearance and image quality of the HR domain Y is transferred to a LR input video frame $x_{t}$ , we resort to adversarial training, given its established effectiveness for domain mapping problems [16]. Our model consists of a generator G , shown in Fig. 2, and a PatchGAN discriminator D [17] for which the following standard adversarial objective is optimized:

(1) $$L_{GAN}=E_{y∼Y}\log D(y)+E_{x∼X}\log(1-D(G(x)))$$

By minimizing this loss, G learns to generate video frames ${\hat{y}}_{t}$ that are indistinguishable by D from real images y∈Y . It is also noted that the generator in the above equation receives as input sequences of frames x∈X and the discriminator operates over single frames. This choice is discussed next.

### 2.4 Incorporating temporal information in the generator

To incorporate temporal information we condition the generation of ${\hat{y}}_{t}$ on a time window of the input video sequence x . More specifically, given a sequence of N consecutive LR iOCT frames $x_{\left[t-N:t\right]}$ , we obtain the corresponding super-resolved frame as ${\hat{y}}_{t}=G(x_{\left[t-N:t\right]})$ . In this work, G (shown in Fig. 2) is a typical bidirectional recurrent network that adopts essential components from the foundational work of BasicVSR [29]. Recurrent VSR networks, as BasicVSR, have showcased excellent performance in natural video super-resolution by spatially increasing the input’s pixel resolution (by a factor of: 2 or 4 ), thus we integrate several key modules from bidirectional propagation, flow estimation and warping modules within our proposed framework, to incorporate the temporal information. Importantly, while [29] proposes these architectural designs when pixel-level supervision is available, we explore their applicability and importance in the more challenging task where no such paired data are available and the former also includes a domain mapping component (Sec. 2.2).

**Fig. 3.**
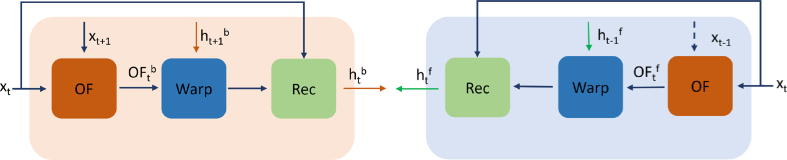
Bidirectional feature propagation: Backward Branch (Left), Forward branch (Right). OF , Warp , Rec refer to optical flow estimation, spatial warping and reconstruction modules respectively.

First, the input sequence $x_{\left[t-N:t\right]}$ is downsampled and subsequently passed through the backward propagation branch. Backward propagation includes optical flow estimation, spatial warping and reconstruction modules. Particularly, as shown in Fig. 3 (left), during the backward propagation, optical flow is estimated between $x_{t+1}$ and $x_{t}$ and is utilized for spatial alignment by performing warping on the propagated features $h_{t+1}^{b}$ from the adjacent t+1 frame. The aligned features are then passed through multiple residual blocks (reconstruction module) for refinement as commonly used in super-resolution networks [15]. The equations that describe the functioning of the backward branch are the following:

(2) $$OF_{t}^{b}=OF(x_{t},x_{t+1})$$

(3) $$h_{t}^{b}=Rec(Warp(OF_{t}^{b},h_{t+1}^{b}),x_{t})$$

where OF , Warp , Rec denote optical flow estimation, warping and reconstruction modules respectively. b refers to backward propagation and h pertains to the propagated features.

Subsequently, the downsampled $x_{\left[t-N:t\right]}$ sequence is fed into the forward propagation branch (see Fig. 3 (right)) which contains similar components as those in backward propagation. However, in the forward branch, the input sequence is utilized in reverse order. Similar set of equations applies to the forward case:

(4) $$OF_{t}^{f}=OF(x_{t},x_{t-1})$$

(5) $$h_{t}^{f}=Rec(Warp(OF_{t}^{f},h_{t-1}^{f}),x_{t})$$

where f refers to forward branch.

Finally, we perform aggregation and upsampling operations to the outputs obtained from both backward and forward branches to generate a HR output, denoted ${\hat{y}}_{t}$ , for every $x_{t}$ :

(6) $${\hat{y}}_{t}=Up(Aggr(h_{t}^{f},h_{t}^{b}))$$

where Aggr and Up pertain to the Aggregation and Upsampling modules.

### 2.5 Preserving structural information

The adversarial loss alone does not guarantee that structural information in $x_{t}$ , such as the exact location and shape of surgically targeted retinal layers is preserved during this mapping. Thus, we require an additional constraint that explicitly enforces spatial and structural consistency between $x_{t}$ and ${\hat{y}}_{t}$ .

To this end, we apply a multi-layer patch-wise contrastive loss [30] that enforces that the local and global spatial structure of the input frame $x_{t}$ is preserved in ${\hat{y}}_{t}$ by maximizing the mutual information between corresponding input and generated patches.

**Fig. 4.**
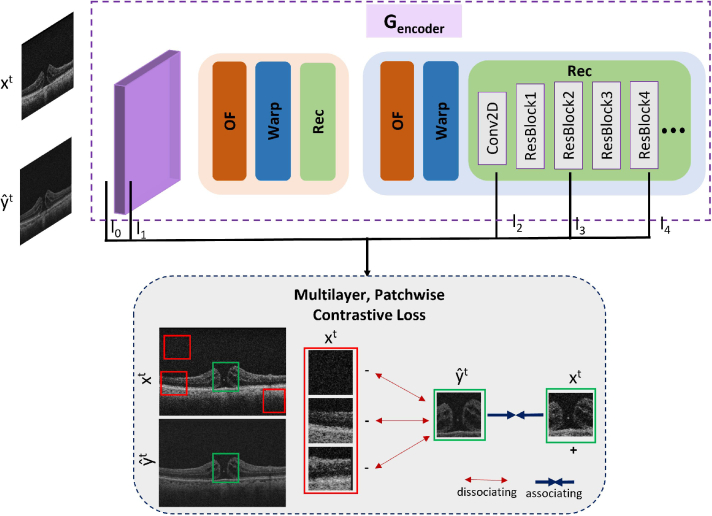
Multilayer Patchwise Contrastive Loss using part of the video super-resolution generator as encoder. Both x and ${\hat{y}}_{t}$ are encoded in features. $l_{0},l_{1},l_{2},l_{3},l_{4}$ are the multiple layers that we extracted features from. The features of a generated output patch should be closer to the features of the corresponding patch in the input image (associating) compared to random patches from different locations (dissociating).

To obtain such features from a common embedding space, both the input video x and the generated images $\hat{y}$ are passed through the generator to extract features from multiple layers indexed by L = { $l_{0},l_{1},l_{2},l_{3},l_{4}$ } (denoted by black lines in Fig. 4), comprising both early high-resolution layers that encode local information and deeper lower-resolution layers that produce more contextualized features.

Then, for each layer l , we randomly sample a subset of the features extracted by the generator from $x_{\left[t-N:t\right]}$ denoted as $\{u_{i}{\}}^{l}$ . We also select their spatially corresponding counterparts $\{v_{i}{\}}^{l}$ from features extracted by the generator from ${\hat{y}}_{t}$ and treat them as the positive samples of $\{u_{i}{\}}^{l}$ . Finally, as negatives we sample a subset of the features extracted by the generator from $x_{\left[t-N:t\right]}$ from different spatial locations than those of the two previous sets, denoted as $\{n_{i}{\}}^{l}$ . Then the contrastive loss is defined as:

(7) $$L_{Contr}=-\sum_{l\in L}\sum_{i=1}^{P^{\left(l\right)}}\log\frac{\exp(u_{i}\cdot v_{i}/\tau )}{\exp(u_{i}\cdot v_{i}/\tau )+\sum_{k=1}^{K^{\left(l\right)}}\exp(u_{i}\cdot n_{k}/\tau )}$$

where $P^{\left(l\right)}=|\{u_{i}{\}}^{l}|=|\{v_{i}{\}}^{l}|$ , $K^{\left(l\right)}=|\{n_{i}{\}}^{l}|$ ( |⋅| being cardinality) and τ=0.07 .

To further encourage structure consistency between input and output, we use a perceptual loss [15] $L_{Perc}$ . Our complete objective then becomes:

(8) $$L_{total}=\lambda_{1}L_{GAN}+\lambda_{2}L_{Contr}+\lambda_{3}L_{Perc}$$

where $\lambda_{1}$ , $\lambda_{2}$ and $\lambda_{3}$ denote the weights assigned to $L_{GAN}$ , $L_{Contr}$ and $L_{Perc}$ losses respectively.

### 2.6 Implementation details

To train the recurrent VSR model, we used a strided 1×1 convolution for downsampling and the pre-trained SPyNet [31] as optical flow estimation module. For the reconstruction module, we used 10 residual blocks of 128 feature channel with instance normalization [32] and ReLU non-linearities. For aggregation, we used feature concatenation, and for upsampling pixel-shuffle [33] and hyperbolic tangent function (tanh) as last layer in the generator to produce output between [−1,1] .

The learning rate for all the modules is set to $10^{-4}$ adopting linear decay policy after 50 epochs. Our model is trained using Adam optimizer and a batch size of 8 for a total of 200 epochs. We used NVIDIA Tesla V100 SXM3 32 GB for our experiments.

In order to calculate the multi-layer, patch-based contrastive loss, we extract features from 5 layers: the grayscale pixels, the downsampling convolution, the first convolution, the second and the fourth residual block of the reconstruction module. This approach aligns with the application of the contrastive loss, as outlined in the foundational work [30], as well as the utilization of the VGG loss, as demonstrated in [15]. Both methods employ layers ranging from pixel-level up to a deep convolutional layer. We base our approach on the same rationale.

Furthermore, we choose $\lambda_{1}=1$ , $\lambda_{2}=20$ and $\lambda_{3}=1$ . The coefficients were experimentally determined, aligning with the methodology outlined in the original paper [30], wherein they selected $\lambda_{1}=1$ and $\lambda_{2}=10$ . Through our own exploration, we identified that the application of an increased $\lambda_{2}$ , specifically $\lambda_{2}=20$ , serves to establish higher preservation of structural information between the input and the generated output.

## 3. Experiments

In this section, we report the results derived from the quantitative analysis performed to assess the efficacy of our approach. We employed nine different no-reference image quality metrics for evaluation purposes. Additionally, we conducted ablation studies to highlight the key contributions of our methodology.

### 3.1 Quantitative analysis

We conducted an evaluation to assess the image quality improvement between real iOCT video frames and those generated by our iOCT video super-resolution methodology video frames. We utilized a test set comprising a total of 1738 iOCT video sequences, extracted from iOCT vitreoretinal surgery videos of 9 patients, all of whom were not included in the training set. Since aligned ground truth HR video sequences are not available, we used nine different no-reference IQA metrics to extensively assess the image quality. These metrics include Fréchet Inception Distance (FID) [34], Kernel Inception Distance (KID) [35], perceptual loss function $\ell_{feat}$ [25], Global Contrast Factor (GCF) [36], Fast Noise Estimation (FNE) [37], Precision and Recall (P&R) [38] and Density and Coverage (D&C) [39]. To gauge the impact of our SR method, we calculated |Δ GCF | , which quantifies the absolute difference in the mean GCF values between SR images and preOCT images as well as |Δ FNE | , which measures the absolute difference in the mean FNE values between SR and preOCT images. These evaluation metrics enable a comprehensive assessment of the image quality enhancement achieved by our methodology in the absence of available ground truth HR video sequences.

Table 1 summarizes the results of our quantitative analysis. Our unpaired video super-resolution approach demonstrates superior performance compared to all the other iOCT super-resolution methods, ranking first in six out of nine metrics. We conducted comparisons with the state-of-the-art [25–27] and also with the single-image super-resolution model using contrastive learning (SRCUT) as proposed in the original paper [30]. It is important to note that CycleGAN [16], the state-of-the-art two-sided image-to-image translation method, failed to generate images of satisfactory quality. Therefore, we excluded it from the comparisons.

**Table 1.** Quantitative analysis on the generated HR OCT images comparing different single-image SR approaches with our VSR approach. Arrows show whether higher/lower is better.

	FID (↓)	KID (↓)	$\ell_{feat}(↓)$	|Δ GCF |(↓)	|Δ FNE |(↓)	P&R (↑)	D&C (↑)
iOCT	156.44	0.16	513.97	2.36	8.04	0.85/0.63	0.15/0.44

[25]	149.83	0.137	481.76	1.94	7.53	0.86/0.65	0.2/0.49
[27]	114.09	0.086	398.73	0.76	3.99	0.88/0.70	0.24/0.61
[26]	99.46	0.082	354.01	0.77	3.75	**0.97**/0.79	0.46/0.79
SRCUT	86.01	0.057	325.02	0.51	**0.06**	0.94/**0.86**	0.38/0.82

Ours	**74.85**	**0.047**	**312.71**	**0.50**	0.12	0.95/0.80	**0.48**/ **0.89**

**Fig. 5.**
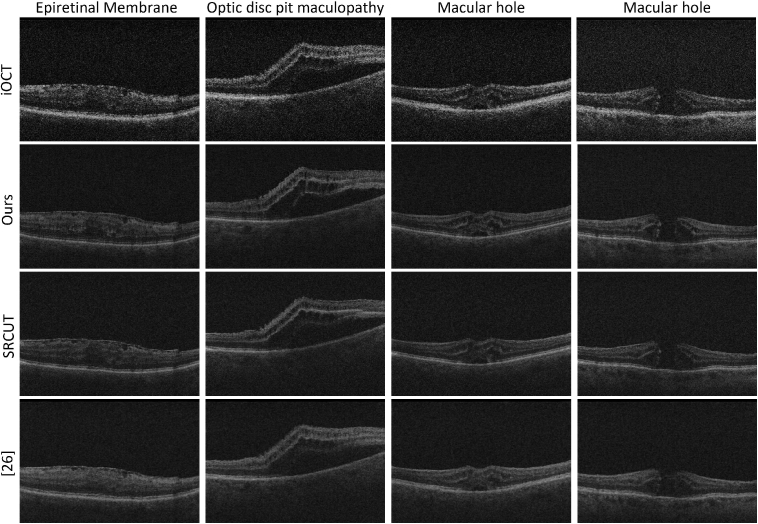
Visual comparison between different SR methods. From top to bottom: LR iOCT images, SR using our UVSR approach, SRCUT and SR using [26]. See also **Visualization 1, Visualization 2, Visualization 3, Visualization 4, Visualization 5, Visualization 6, Visualization 7 and Visualization 8** for video comparisons.

**Fig. 6.**
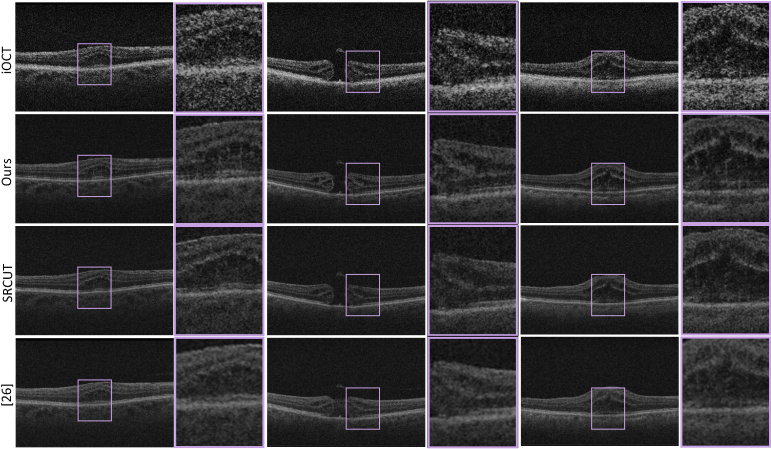
Visual comparison of different SR methods. From top to bottom: LR iOCT images, SR using our UVSR approach, SRCUT and SR using [26].

**Fig. 7.**
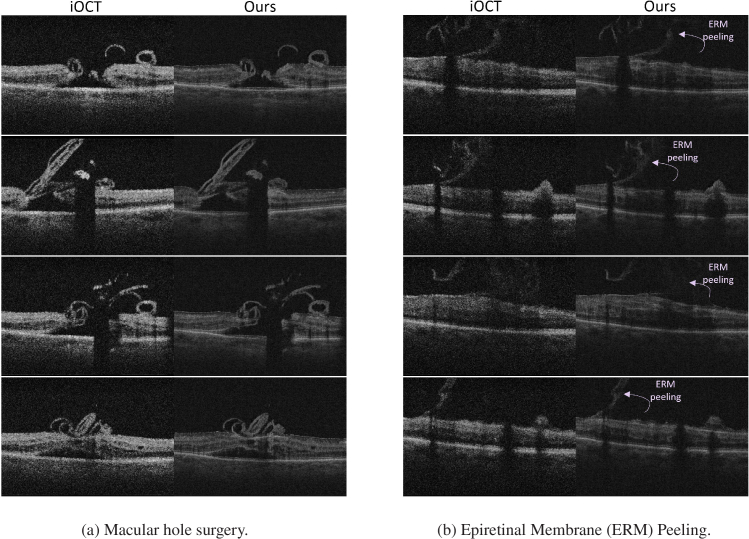
Visual results of our proposed method in challenging scenarios encountered during vitreoretinal surgeries. (a) Macular hole: Our method effectively preserves the original structural elements and maintains the interaction between the retina and surgical tools as seen in the input. (b) ERM Peeling: The output distinctly illustrates the presence of epiretinal membrane (ERM) in unpeeled retinal areas, while accurately depicting the absence of ERM in the peeled regions. Remarkably, the model achieves an acceptable level of generalization, even when faced with inputs that lie outside the usual realm of its training and testing data.

Our approach showcases remarkable performance in perceptual metrics such as FID, KID, $\ell_{feat}$ . Lower FID values indicate lower distance and, consequently, higher similarity between the distributions of the generated SR and real preOCT images in a deep feature domain, encompassing both low-level and high-level features. Therefore, our methodology generates SR iOCT images of the highest quality as they closely resemble the images of preOCT domain which contains high levels of spatial details and diagnostic utility (see also Fig. 5 and Fig. 6 and Fig. 7). Furthermore, the KID values, which offer a similar but more advantageous than FID metric along with the $\ell_{feat}$ values, indicate highest level of quality enhancement achieved by our method, concerning perceptual quality. As generated images highly resemble preOCT images, they are characterized by finer and high-frequency details avoiding noticeable blurriness and discontinuities in anatomical structures. This is attributed to the establishment of a stable adversarial training, which has shown remarkable effectiveness in domain mapping problems. Moreover, it is noteworthy that the omission of L1 and L2 loss functions from training likely contributed to the reduction of blurriness of the results as they are recognized for their potential to introduce blurriness into the generated output.

Moreover, recognising that perceptual metrics may not be able to capture and assess low-level characteristics, we provide an analysis of the |ΔGCF| and |ΔFNE| values, where our method achieves the lowest (best) and the second lowest values, respectively. This divergence appears reasonable as noise and contrast values do not necessarily depend on each other or have to follow the same trend. Notably, the worse | ΔFNE | value of our method could be attributed to the FNE’s inclination to identify thin lines as noise, as stated in [37]. Nevertheless, our method demonstrate improved | ΔGCF | and | ΔFNE | values compared to previous methods ensuring that the contrast extracted from various resolution levels (GCF) and the noise levels (FNE) in SR images closely resemble the corresponding levels in the HR preOCT images, which represent the highest quality images in our dataset.

Additionally, to address the limitations of using single-number perceptual metrics, such as FID, in separating fidelity and diversity, both significant aspects of generative models’ quality, we utilize precision and recall (P&R) metrics. Our method achieves the second highest (best) values in both metrics indicating that our generated SR iOCT images highly resemble the real preOCT images (P) while covering their full variability (R).

Similarly, we incorporate Density and Coverage (D&C) metrics, an improved version of P&R, in our evaluation. Our method attains the highest D and C values among other SR models, further supporting the capability of our network to generate images with high fidelity and diversity concerning the HR domain.

### 3.2 Human evaluation study

To further validate our video super-resolution pipeline, we performed a human evaluation study. Our survey included 20 pairs of LR and generated through our methodology images, randomly selected from the test set. We asked 6 retinal dOCTors/surgeons to evaluate these image pairs by assigning a score between 1 (strongly disagree) and 5 (strongly agree) on the following questions:

**Q1**: Can you notice an improvement in the delineation of RPE/Bruchs vs. IS/OS junction in the generated image? (**A1**: 4.58 ± 0.29)
**Q2**: Do you agree that the generated image does NOT contain artificially hallucinated structures? (**A2**: 4.52 ± 0.05)
**Q3**: Can you notice an improvement in the delineation of the ILM vs. RNFL in the generated image? (**A3**: 4.38 ± 0.22)

The responses, denoted as **A1**, **A2**, **A3**, each accompanied by their respective mean values and standard deviations, demonstrate that the clinicians recognized the improved delineation of RPE vs IS/OS junction (Q1), the absence of artificially hallucinated structures (Q2) and the enhanced delineation of ILM vs RNFL (Q3). Compared to the qualitative results reported in [27], our analysis indicates greater lever of preference and agreement among clinicians regarding important characteristics of our generated images. Across the three questions, we note a substantial elevation in the mean value exceeding 0.5 compared to the results in [27]. Visual results are shown in Fig. 5 and Fig. 6, complementing the findings of our survey.

### 3.3 Importance of different loss terms for iOCT VSR

**Fig. 8.**
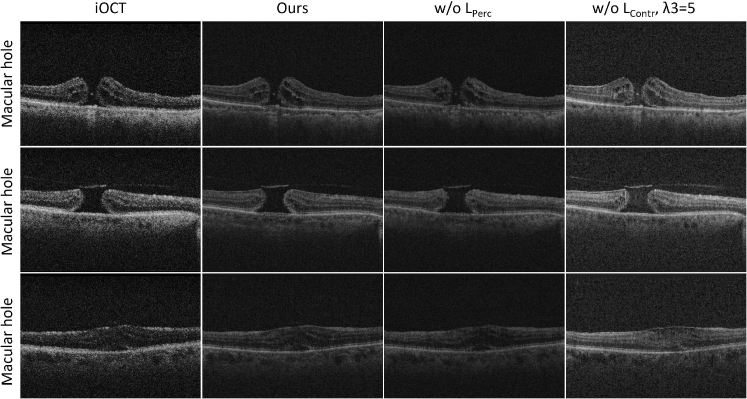
Visual comparison between different training strategies. From left to right: LR iOCT images, ours (using both $L_{Contr}$ and $L_{Perc}$ ), ours w/o $L_{Perc}$ and ours w/o $L_{Contr}$ ( λ3=5 ).

To study the importance of each of the 3 terms of the overall loss function (Eq. (8)) that was used for training the proposed unsupervised video super-resolution model, we conducted a series of ablation experiments, each evaluating the effect of removing a loss term. The results are presented in Table 2 (Fig. 8). We have omitted the $\ell_{feat}$ and P&R values due to spatial constraints. Instead, we have included FID and D&C values as they represent similar aspects of the evaluation.

**Table 2.** Quantitative analysis on the generated HR OCT images using different training strategies.

	FID (↓)	KID (↓)	|Δ GCF |(↓)	|Δ FNE |(↓)	D&C (↑)
w/o $L_{Perc}$	85.33	0.056	1.04	0.78	**0.52**/0.85
w/o $L_{Contr}(\lambda_{3}=1)$	275.48	0.314	0.69	3.30	0.08/0.07
w/o $L_{Contr}(\lambda_{3}=5)$	79.31	0.052	2.15	5.22	0.34/0.76
w/o $L_{Contr}(\lambda_{3}=10)$	101.08	0.078	4.21	9.07	0.25/0.70
w/o $L_{GAN}$	153.69	0.161	2.38	8.04	0.16/0.48

Ours	**74.85**	**0.047**	**0.50**	**0.12**	0.48/ **0.89**

Removing the perceptual loss (w/o $L_{Perc}$ ) leads to a moderate drop on all metrics. Removing the (w/o $L_{Contr}$ , $\lambda_{3}=1$ ) contrastive term and keeping the perceptual term’s weight in its default value ( $\lambda_{3}=1$ ) results in significantly worse super-resolution results, as reported in Table 2.

Furthermore, removing the contrastive loss term and adjusting the weights for the perceptual loss, (w/o $L_{Contr}$ , $\lambda_{3}=5$ and w/o $L_{Contr}$ , $\lambda_{3}=10$ ), improves performance but still results in slightly worsen perceptual quality metrics (as measured by FID and KID). Importantly, in these cases the obtained |Δ GCF | and |Δ FNE | are higher indicating that the contrast and noise levels of the preOCT are better captured by the model when using our complete loss function ("ours"). This observation highlights the importance of contrastive loss in maintaining consistent perceptual quality as well as contrast and noise levels.

Finally, removing the adversarial loss term (w/o $L_{GAN}$ ) significantly impairs performance w.r.t to all metrics, hinting that it is crucial for generating images that capture both the high and low-level aspects of the HR preOCT domain.

### 3.4 Importance of aggregating temporal information

In this section, we highlight the importance of using multiple images with feature propagation between neighbouring frames for iOCT super-resolution. We initially perform inference of our model while setting the output of the feature warping module (depicted as the output of blue box denoted as ’Warp’ in Fig. 3) equal to zero. The rational behind this ablation is to assess whether the reconstruction module can generate high quality images without utilizing warped features from preceding frames. As reported in Table 3, in the absence of feature propagation from previous frames, the model ’w/o feat_prop’ achieves in worse results on all metrics.

**Table 3.** Ablation study on the importance of aggregating temporal information.

	Frames	FID (↓)	KID (↓)	|Δ GCF |(↓)	|Δ FNE |(↓)	D&C (↑)
w/o feat_prop	5	120.24	0.107	2.25	22.12	0.16/0.58

ours-2frames	2	97.54	0.058	0.55	0.18	0.38/0.85
ours-3frames	3	87.75	0.059	1.06	12.16	0.38/0.85
ours-4frames	4	78.17	0.051	**0.10**	2.36	0.43/0.84

Ours	5	**74.85**	**0.047**	0.50	**0.12**	**0.48**/ **0.89**

**Fig. 9.**
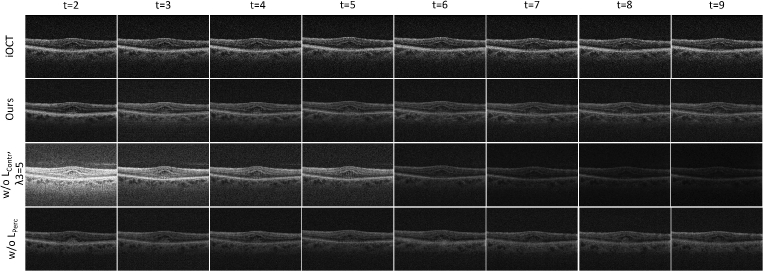
Visual comparison between different training strategies across varying input sequence lengths. From left to right: different timestamps of the input sequence. From top to bottom: LR iOCT images, ours (using both $L_{Contr}$ and $L_{Perc}$ ), ours w/o $L_{Contr}$ ( λ3=5 ) and ours w/o $L_{Perc}$ .

Furthermore, to explore the effect of aggregating temporal information on the ultimate super-resolution outcome, we vary the number of input frames. As shown in Table 3 and $2^{nd}$ row in Fig. 9, our model exhibits a gradual enhancement in performance with the inclusion of additional frames in the input sequence particularly evident in perceptual metrics such as FID, KID as well as the D&C metrics. However, concerning |Δ GCF | and |Δ FNE | , we observe that these values do not exhibit a monotonic improvement with the inclusion of more frames. This observation can be attributed to the fact that our recurrent network was trained on sequences of five frames and has learned to capture and utilize the temporal context within that specific sequence. As a result, during inference, when we reduced the sequence length, we lose some of the contextual information potentially leading to a negative impact on the network’s ability to make accurate predictions.

**Fig. 10.**
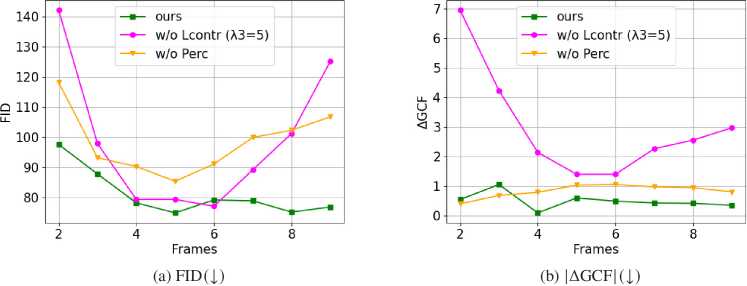
Ablation on temporal information: Graphs illustrating the performance of our methodology with contrastive loss (green curve), without contrastive loss (w/o $L_{Contr}$ ( λ3=5 )) (magenta curve) and without perceptual loss (w/o $L_{Perc}$ ) (orange curve) across varying input sequence lengths in terms of image quality metrics.

We also investigated whether our trained model can effectively handle longer image sequences (ranging from 6 to 9 frames) compared to the different training strategies as shown in Fig. 9. The FID and |Δ GCF | values for each method are presented in the graphs 10(a) and 10(b), respectively. In Fig. 10(a), we observe that our method (green curve), utilizing the contrastive loss, consistently generates images of relatively similar quality as indicated by the relatively constant FID values, across different numbers of input frames. On the contrary, when trained without the constrastive loss (magenta curve), we achieved acceptable results (low FID) solely when using image sequence of a length close to the training (5) frames while higher or lower sequence lengths resulted in worse performance (high FID). Similarly, removing the perceptual loss (orange curve), leads to higher FID values degrading the perceptual quality of the generated images across time.

Similarly, regarding |Δ GCF | , our approach (green) maintains the contrast values at consistent levels across different frames. Analogous trend is observed in the orange curve, which was trained solely with contrastive loss. Conversely, our model trained without contrastive loss exhibited high |Δ GCF | values for frames lower than 4 and higher than 7.

These findings further reinforce the efficacy or our methodology, particularly in handling longer input sequences. According to these graphs, our methodology generates consistently images of high quality particularly in handling image sequences of varying lengths while preserving image quality and contrast characteristics. The integration of contrastive loss assists the generator to learn intermediate representations that encapsulate meaningful semantic or structural information, thereby facilitating the aggregation of propagated features and the reconstruction of high-quality images. Particularly for longer input sequences, the generator is able to effectively propagate and aggregate long-term information. On the contrary, our model trained without contrastive loss, exhibits signs of overfitting, as it may have become overly reliant to using the entire 5-image context for predictions and may face challenges when that context is reduced, leading to decline in performance.

At this point, we would like to note that the selection of 5 frames is rooted in our decision to reduce the training duration in comparison to lengthier input sequences, thus affording us the opportunity to undertake an expanded number of experiments to comprehensively assess our work’s efficacy. It is important to acknowledge that the adoption of extended image sequences for training purposes to capture long-term information remains a promising avenue for future investigation.

### 3.5 Comparison with denoising methods

In this section, we undertake a comparative analysis of our super-resolution method against conventional denoising filter in order to illustrate the denoising efficacy of our work, which forms part of the broader objective of image quality enhancement.

For evaluation, we selected two state-of-the-art speckle reduction methods for OCT images: Non-local means (NLM) [40] and Block-matching and 3D filtering (BM3D) [41]. These methods have been evaluated in previous studies [8,42–44] for their denoising capabilities. The NLM implementation, provided by scikit-image [45], was employed and we conducted tests with two variations: one utilizing estimated noise standard deviation (NLM ( $\tilde{\sigma }$ )) and one without incorporating such estimation (NLM). Additionally, the BM3D approach was assessed using experimentally defined values of σ=0.05 and σ=0.1 as shown in the accompanying Table 4.

**Table 4.** Quantitative analysis on the generated HR OCT images using conventional denoising filters.

	FID (↓)	KID (↓)	|Δ GCF |(↓)	|Δ FNE |(↓)	D&C (↑)
BM3D( σ=0.05 )	225.99	0.252	**0.23**	2.62	0.09/0.29
BM3D( σ=0.1 )	247.14	0.276	0.72	4.96	0.03/0.17
NLM	176.35	0.221	1.16	0.76	0.07/0.24
NLM( $\tilde{\sigma }$ )	227.65	0.268	0.77	1.02	0.09/0.31

Ours	**74.85**	**0.047**	0.50	**0.12**	0.48/ **0.89**

**Fig. 11.**
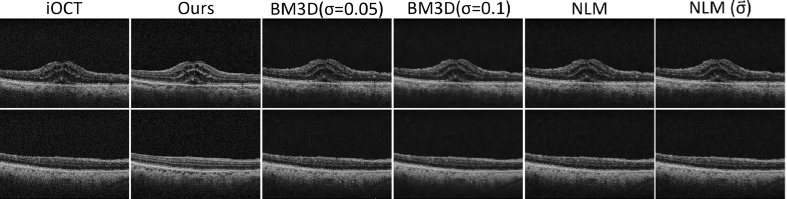
Visual comparison between our method and different conventional denoising approaches. From left to right: LR iOCT images, ours method, denoising using BM3D ( σ=0.05) , BM3D( σ=0.1 ), NLM and NLM ( $\tilde{\sigma }$ ).

Based on the data presented in Table 4 (see also Fig. 11), it is evident that denoised images obtained through conventional techniques do not yield images of comparable quality to those in the preOCT domain. Although these filters may indeed succeed in effectively reducing noise, they are unable to furnish valuable information regarding retinal layers and other crucial structures pertinent to vitreoretinal surgeries, a feat accomplished by our super-resolution method.

## 4. Conclusion

In this study, we proposed an unpaired video super-resolution approach for intra-operative Optical Coherence Tomography (iOCT) videos acquired during vitreoretinal surgeries. Through extensive quantitative analysis, ablation studies and a qualitative analysis by expert clinicians, we demonstrated that our approach can effectively enhance the iOCT image quality achieving a new state-of-the-art performance. Furthermore, we showed that video super-resolution models when trained with multilayer patchwise contrastive loss can effectively aggregate temporal information even in an unpaired scenario.

Potentially, our approach may be applicable in different intra-operative modalities, such as ultrasound and intra-operative MRI as well as research in 3D/4D OCT. Particularly, in the context of 3D/4D OCT, our work has the potential to surpass the outcomes reported in existing studies [43,46], as we utilize pre-operatively acquired OCT scans as HR domain to train our super-resolution network. These scans represent more than merely denoised OCT data that are used in [43,46]; rather, they have undergone comprehensive offline processing, thereby furnishing scans of high-fidelity information and diagnostic utility. Furthermore, the underlying architecture of our recurrent video super-resolution algorithm, which leverages temporal information from a sequence of images, bears the potential to increase the consistency between the generated outputs of adjacent 2D frames within the 3D OCT and thus contribute to an elevation in the overall consistency of the volumetric representation.

However, we acknowledge that our work has several limitations. First, our method exhibits slower runtime speed compared to existing works that perform single-image super-resolution. In our research, we chose to focus on the architectural innovation and training strategy to address a critical gap in the field of unpaired medical video super-resolution. To the best of our knowledge, this study is the first to address the application of video super resolution between unpaired datasets in the medical imaging domain. Moreover, the absence of pre-operative OCT images that illustrate interactions with surgical instruments, a phenomenon quite common during surgeries and iOCT scans, could potentially impact the performance of our model. Additionally, the unavailability of pixel-wise ground truth data poses constraints on our ability to incorporate spatial and temporal reference-based metrics that could further support the evaluation of our work.

In conclusion, our work can be used as a strong foundation for future research and optimization efforts. Our future work will explore improvements of our method’s computational efficiency, incorporation of tool interactions within the pre-operative domain, extension of input sequences capturing long-term dependencies, utilization of additional spatial and temporal metrics and ultimately deployment of our iOCT video super-resolution approach into the clinic.

## Data Availability

Data underlying the results presented in this paper are not publicly available at this time but may be obtained from the authors upon reasonable request. There are parallel endeavours to make them public.
