# Unpaired intra-operative OCT (iOCT) video super-resolution with contrastive learning: erratum

**DOI:** 10.1364/BOE.555423

**Published:** 2025-01-30

**Authors:** Charalampos Komninos, Theodoros Pissas, Blanca Flores, Edward Bloch, Tom Vercauteren, Sébastien Ourselin, Lyndon Da Cruz, Christos Bergeles

**Affiliations:** 1 School of Biomedical Engineering & Imaging Sciences, King’s College London, SE1 7EU, London, UK; 2 Moorfields Eye Hospital, EC1V 2PD, London, UK; 3 Institute of Ophthalmology, University College London, EC1V 9EL, London, UK

## Abstract

Errata are presented to amend the values presented in results section and corresponding figures in our published manuscript [Biomed. Opt. Express 15, 772 (2024)10.1364/BOE.50174338404298 PMC10890864]. These corrections do not alter the main findings of our publication.

Due to incosistencies in the number of iOCT image sequences and the number of frames per sequence utilised for evaluation across different approaches, we provide corrections to the tables and figures in [1] as follows, with the corrected method(s) indicated in parentheses: Table 1 ([2], Ours), Fig. 8 (4th column), Table 2 (w/o $L_{Contr}(\lambda_{3}=1),(\lambda_{3}=5),(\lambda_{3}=10)$ , w/o $L_{GAN}$ , Ours), Table 3 (ours-2frames, Ours), Fig. 10 (FID and |ΔGCF| graphs) and Table 4 (NLM, NLM( $\tilde{\sigma }$ ), Ours).

**Fig. 8.**
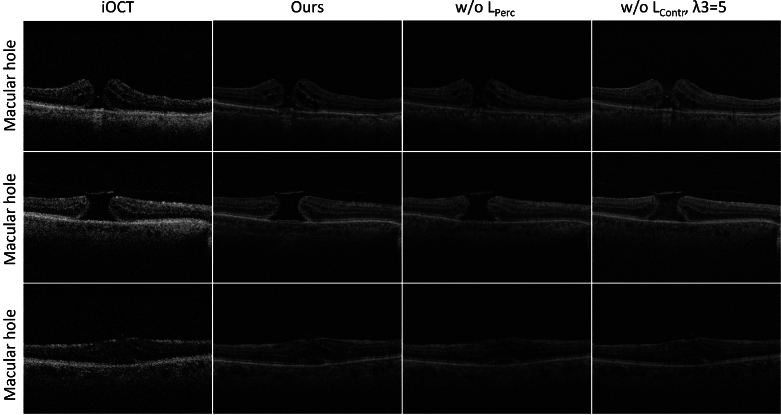
Visual comparison between different training strategies. From left to right: LR iOCT images, ours (using both $L_{Contr}$ and $L_{Perc}$ ), ours w/o $L_{Perc}$ and ours w/o $L_{Contr}\;(\lambda 3=5)$ .

**Fig. 10.**
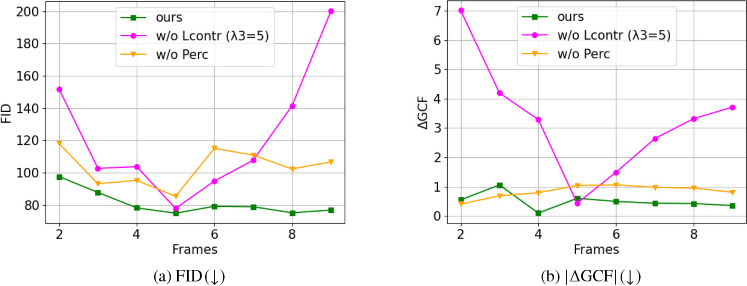
Ablation on temporal information: Graphs illustrating the performance of our methodology with contrastive loss (green curve), without contrastive loss (w/o $L_{Contr}\;(\lambda 3=5)$ ) (magenta curve) and without perceptual loss (w/o $L_{Perc}$ ) (orange curve) across varying input sequence lengths in terms of image quality metrics.

**Table 1.** Quantitative analysis on the generated HR OCT images comparing different single-image SR approaches with our VSR approach. Arrows show whether higher/lower is better.

	FID(↓)	KID(↓)	$\ell_{feat}(↓)$	|ΔGCF|(↓)	|ΔFNE|(↓)	P&R(↑)	D&C(↑)

iOCT	156.44	0.164	513.97	2.36	8.04	0.85/0.63	0.15/0.44
[2]	158.00	0.156	436.84	2.70	2.76	0.70/0.45	0.11/0.45
[3]	114.09	0.086	398.73	0.76	3.99	0.88/0.70	0.24/0.61
[4]	99.46	0.082	354.01	0.77	3.75	**0.97**/0.79	0.46/0.79
SRCUT	86.01	0.057	325.02	**0.51**	**0.06**	0.94/**0.86**	0.38/0.82

Ours	**73.41**	**0.045**	**309.76**	0.61	0.12	0.96/0.81	**0.48/0.85**

**Table 2.** Quantitative analysis on the generated HR OCT images using different training strategies.

	FID(↓)	KID(↓)	|ΔGCF|(↓)	|ΔFNE|(↓)	D&C(↑)
$\text{w/o}\;L_{Perc}$	85.33	0.056	1.04	0.78	0.52/0.85
$\text{w/o}\;L_{Contr}(\lambda_{3}=1)$	88.49	0.063	0.54	2.16	0.55/0.88
$\text{w/o}\;L_{Contr}(\lambda_{3}=5)$	77.82	0.047	**0.44**	0.39	0.46/**0.91**
$\text{w/o}\;L_{Contr}(\lambda_{3}=10)$	78.89	0.046	0.63	0.53	0.47/0.90
$\text{w/o}\;L_{GAN}$	110.44	0.088	0.97	1.12	0.22/0.52

Ours	**75.92**	**0.045**	0.72	**0.04**	**0.56**/0.90

**Table 3.** Ablation study on the importance of aggregating temporal information.

	Frames	FID(↓)	KID(↓)	|ΔGCF|(↓)	|ΔFNE|(↓)	D&C(↑)

w/o feat_prop	5	120.24	0.107	2.25	22.12	0.16/0.58
ours-2frames	2	97.54	0.071	0.55	0.19	0.28/0.70
ours-3frames	3	87.75	0.059	1.06	12.16	0.38/0.85
ours-4frames	4	78.17	0.051	**0.10**	2.36	0.43/0.84

Ours	5	**74.85**	**0.047**	0.61	**0.12**	**0.48**/ **0.89**

**Table 4.** Quantitative analysis on the generated HR OCT images using conventional denoising filters.

	FID(↓)	KID(↓)	|ΔGCF|(↓)	|ΔFNE|(↓)	D&C(↑)
BM3D (σ=0.05)	225.99	0.252	**0.23**	2.62	0.09/0.29
BM3D (σ=0.1)	247.14	0.276	0.72	4.96	0.03/0.17
NLM	230.95	0.276	0.49	0.39	0.09/0.31
NLM $\left(\tilde{\sigma }\right)$	221.88	0.262	0.65	0.95	0.10/0.38

Ours	**74.85**	**0.047**	0.61	**0.12**	**0.48**/ **0.89**

Furthermore, we correct related phrases in the text as follows:

Section 3.1, 2nd paragraph, 2nd sentence is corrected as follows: Our unpaired video super-resolution approach demonstrates superior performance compared to all the other iOCT super-resolution methods, ranking first in five out of nine metrics.

Section 3.1, 4th paragraph, 1st and 2nd sentences are corrected as follows: Moreover, recognising that perceptual metrics may not be able to capture and assess low-level characteristics, we provide an analysis of the |ΔGCF| and |ΔFNE| values, where our method achieves the second lowest (best) values.

Section 3.3, 2nd paragraph, 2nd sentence is corrected as follows: Removing the (w/o $L_{Contr},\lambda_{3}=1$ ) contrastive term and keeping the perceptual term’s weight in its default value $\left(\lambda_{3}=1\right)$ results in worse super-resolution results, as reported in Table 2.

Section 3.3, 3rd paragraph, 1st and 2nd sentences are corrected as follows: Furthermore, removing the contrastive loss term and adjusting the weights for the perceptual loss, (w/o $L_{Contr},\lambda_{3}=5$ and w/o $L_{Contr},\lambda_{3}=10$ ), improves performance but still results in slightly worsen quality metrics. Our method outperforms the w/o $L_{Contr}$ approaches in most of the metrics indicating that the perceptual quality and noise levels of the preOCT are better captured by the model when using our complete loss function ("ours").

Regarding Table 1, after the corrections, our approach (Ours) loses its superiority in |ΔGCF| but remains best in most of the metrics.

Regarding the comparison with the w/o $L_{Contr}$ variations in Table 2, after the corrections our approach (Ours) loses its superiority in |ΔGCF| and Coverage(C) metrics while achieves best value in Density (D). As shown in Table 2, it remains superior in most of the metrics. Particularly, it outperforms the w/o $L_{Contr},\lambda_{3}=1$ and $\lambda_{3}=10$ in 5 out of 6 metrics and the w/o $L_{Contr},\lambda_{3}=5$ in 4 out of 6 metrics. Similarly, concerning the 9 metrics, our method outperforms the w/o $L_{Contr},\lambda_{3}=1$ and $\lambda_{3}=10$ in 7 out of 9 metrics and the w/o $L_{Contr},\lambda_{3}=5$ in 5 out of 9 metrics. In Table 2, the reported values for our approach differ from those in other tables because we use a checkpoint obtained at a later stage of the training. The results reported in all the remaining tables, as well as the qualitative analysis and figures, are derived from the same checkpoint.

The reported corrections do not change the information presented in the abstract, introduction, methods and discussion sections of the original paper, and they do not alter the main conclusions of our manuscript. Therefore, limited corrections are required in the main text.
